# A nanoparticle vaccine displaying varicella-zoster virus gE antigen induces a superior cellular immune response than a licensed vaccine in mice and non-human primates

**DOI:** 10.3389/fimmu.2024.1419634

**Published:** 2024-07-16

**Authors:** Yuanyuan Li, Siyu Tian, Yuanbao Ai, Zhulong Hu, Chao Ma, Meijuan Fu, Zhenqian Xu, Yan Li, Shuyun Liu, Yongjuan Zou, Yu Zhou, Jing Jin

**Affiliations:** Patronus Biotech Co. Ltd., Guangzhou, China

**Keywords:** shingles vaccine, gE, NP, VLP, tag/catcher system, CMI, herpes zoster

## Abstract

Herpes zoster (HZ), also known as shingles, remains a significant global health issue and most commonly seen in elderly individuals with an early exposure history to varicella-zoster virus (VZV). Currently, the licensed vaccine Shingrix, which comprises a recombinant VZV glycoprotein E (gE) formulated with a potent adjuvant AS01B, is the most effective shingles vaccine on the market. However, undesired reactogenicity and increasing global demand causing vaccine shortage, prompting the development of novel shingles vaccines. Here, we developed novel vaccine candidates utilising multiple nanoparticle (NP) platforms to display the recombinant gE antigen, formulated in an MF59-biosimilar adjuvant. In naïve mice, all tested NP vaccines induced higher humoral and cellular immune responses than Shingrix, among which, the gEM candidate induced the highest cellular response. In live attenuated VZV (VZV LAV)-primed mouse and rhesus macaque models, the gEM candidate elicited superior cell-mediated immunity (CMI) over Shingrix. Collectively, we demonstrated that NP technology remains a suitable tool for developing shingles vaccine, and the reported gEM construct is a highly promising candidate in the next-generation shingles vaccine development.

## 1 Introduction

Varicella-zoster virus (VZV) is a neurotropic human alphaherpesvirus that causes two main categories of diseases. Primary infection results in varicella (chickenpox), which predominantly occurs in children and is characterized by a generalized vesicular pruritic rash on the trunk, head, and face (1). The reactivation of latent virus due to age-related immune system decline causes herpes zoster (HZ), also known as shingles, with Postherpetic neuralgia (PHN) as the most common complication (2). HZ affects approximately one-third of the world’s population, and its risk increases with age (3–5). It was estimated that 14.9 million HZ cases occurred globally in people aged ≥ 50 in 2020 and were expected to increase to 17.0 million in 2025 and 19.1 million cases by 2030 due to the global trend of population ageing (6).

The humoral response of the immune system is believed to be involved only in controlling the primary VZV infection (7) but not with protection against HZ, as the level of VZV-specific antibodies remains high during life post-infection (8). The VZV-specific cell-mediated immunity (CMI) declines with age (9), correlating to an increased incidence of HZ (10). Later studies have confirmed that VZV-specific CMI is inversely correlated with HZ and is necessary to suppress the reactivation of the latent virus (11–14). Currently, two licensed vaccines are available to prevent shingles in the global market: a live-attenuated vaccine (Zostavax, Merck) and a recombinant protein vaccine (Shingrix, GlaxoSmithKline). Shingrix comprises a recombinant VZV glycoprotein E (gE), the most abundant glycoprotein on the surface of VZV, formulated with a potent adjuvant AS01B (QS-21, monophosphoryl lipid A and liposome). Shingrix elicits a strong VZV-specific CD4 T cell response at least ten times stronger than those from Zostavax (15). Consequently, in pivotal trials, Shingrix demonstrated remarkably better protection (91% overall efficacy) than Zostavax (51% overall efficacy) (16, 17), confirming immunity against gE alone is sufficient to confer protection. gE-specific CMI was then demonstrated in a large-scale cohort study as the clearest correlate of protection (CoP) (14). Although highly effective, there are rising safety concerns about Shingrix regarding the significant number of adverse events (AEs) likely to be associated with reactogenicity from the potent AS01B adjuvant. Pooled safety data analysis from two Shingrix pivotal trials revealed 3.8% grade 3 AEs compared to 0.2% in the placebo group, with pain being the most frequent symptom (18). Additionally, there are also questions regarding the sustainability of QS-21, a natural extract from the tree bark of *Quillaja Saponaria* and a key component in the AS01 adjuvant, which is of limited supply (19) and has been used in various licensed vaccines, including Mosquirix (20), Shingrix (21), Nuvaxovid (22), Arexvy (23) and R21/Matrix-M (24). Therefore, there is a clear need to develop an effective shingles vaccine with improved safety and accessibility.

Nanoparticles (NPs) and Virus-like particles (VLPs) are terminologically similar and sometimes interchangeable. NPs usually originate from natural scaffold or oligomer-forming molecules, while VLPs are composed of natural viral structure proteins (e.g. capsid protein), both of which can self-assemble into particles that resemble native virions in size and structure while remaining non-infectious due to a lack of genetic materials (25). When used as antigen display platforms, the antigens can form an organized array on the surface of these particles, which promotes the activation of B cells by crosslinking multiple B cell receptors (BCRs) (26). NPs and VLPs are also preferentially recognized and internalized by antigen-presenting cells (APCs) such as dendritic cells and macrophages due to their size, leading to more antigen presentation on MHC molecules, which subsequently activates T cells (27). Overall, NPs and VLPs can induce stronger antibody and cellular responses than soluble antigens as already demonstrated by multiple preclinical studies (28–31).

The first natural VLP vaccine was developed against the hepatitis B virus (HBV), and the vaccine is self-assembled by the hepatitis B virus surface antigen (HBsAg) (32). The other natural VLP-based vaccine was approved to protect against human papillomaviruses (HPV), and the vaccine is assembled from the major capsid protein L1 of HPV (33). Both vaccines exhibited >90% efficacy in their corresponding human trials (34, 35) and the success of the HBV and HPV VLP vaccines has driven the development of chimeric particle vaccines, in which the particle serves as an antigen-displaying platform. RTS, S was the first vaccine to exploit VLP as a vaccine carrier, this chimeric VLP was generated by directly fusing malaria antigen circumsporozoite protein (CSP) to HBsAg and co-expressed the fusion protein with native HBsAg in a 1:4 ratio in *Saccharomyces cerevisiae*. The CSP antigen was displayed on the surface of HBsAg-based VLP (36), the vaccine has been proven to have modest efficacy (the protection rate for infants in the first year was 56%) in humans and was eventually approved by WHO (37). Similarly, an improved malaria vaccine R21 was generated, consisting of only CSP-HBsAg fusion protein expressed by *Picha pastoris*. R21 displays a higher number of antigens on the surface of each particle than RTS, S, as a result, it demonstrated a higher efficacy (the protection rate for infants in the first 6 months is 77%) (38). Other chimeric NP or VLP, such as Covifenz (COVLP) developed by Medicago using the genetic fusion method, has also demonstrated high efficacy against SARS-COV-2 in humans (39). SpFN is another chimeric NP vaccine targeting SARS-COV-2, developed by genetically fusing the spike protein of SARS-COV-2 to the ferritin subunit (40). Genetic fusion is not always applicable to all antigens as exogenous protein may interfere with the particle formation. Apart from the traditional chemical conjugation, bio-conjugation technology has advanced fast in recent years to allow easy linkage of antigen protein to the particles. A catcher/tag covalent bond linkage system was developed by splitting *Streptococcus pyogenes*-derived fibronectin-binding protein FbaB (41). LYB001, a SARS-CoV-2 NP vaccine, was generated based on this split protein Tag/Catcher technology (30). The I53−50 NP platform was an icosahedral nanoparticle, formed via co-assembly of two components. The SARS-COV-2 NP vaccine SKYCovione (GBP510) was developed based on this I53−50 NP platform (42). Chimeric NP or VLP vaccines generated by such bio-conjugation technology have proven successful in both preclinical and clinical studies (25).

Maintaining high immunogenicity while reducing the need for potent adjuvants should be a priority for the next-generation shingles vaccine, given that the efficacy of the current market vaccine Shingrix already exceeds 90% (21). It appeared promising to explore the use of NPs and VLPs for displaying an established VZV antigen such as gE while using a modest adjuvant with a good safety and immunogenicity profile. Currently, there are few studies on the development of particle vaccines for shingles. The most relevant study was to display gE peptides on HBc VLP (43), however, this study focused only on the antibody response and lacked the market vaccine control group to make a full assessment of the vaccine.

In this study, we aimed to design a NP or VLP-based shingles vaccine as a standalone or formulated in less potent but safer adjuvants for evaluations in the most up-to-date preclinical models. Bio-conjugation systems and a wide range of vectors were examined for displaying the Shingrix-validated gE antigen, including the catcher/tag isopeptide linkage system as reported previously (44), the I53-50 two-component NP system (45), a computationally designed NP vector (30) named NPM in the present study, the ferritin NP vector (28) and the bacteriophage AP205 VLP vector (46). The evaluation plan included immunogenicity assessments in mice focusing on the gE-specific CMI to down-select the candidates before a non-human primate (NHP) study to provide further proof-of-concept. For consistency purposes, the NP and VLP platforms presented in this study are collectively termed NP.

## 2 Materials and methods

### 2.1 Expression and purification of recombinant gE antigens and nanoparticle carriers

**Figure 1:**
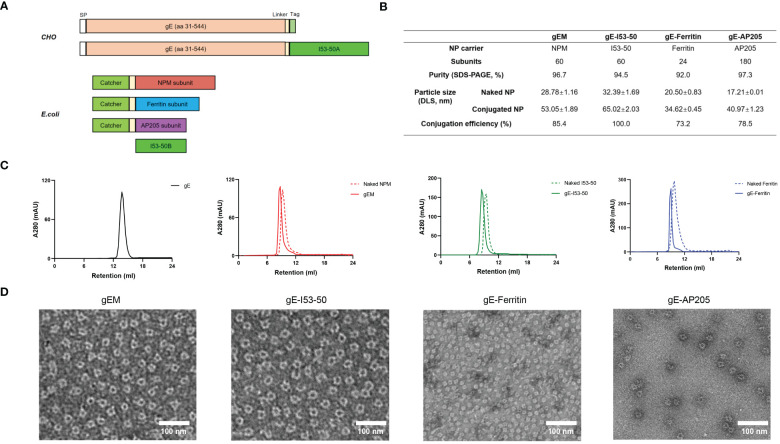
Development and characterization of different gE-NPs. **(A)** Schematic representation of the gE antigens and NP carriers, SP = signal peptide. **(B)** Characterization of different NP carriers and gE-NPs. **(C)** Size exclusion chromatography results (SEC) of the gE antigen, gE-NPs and naked NPs on a Superdex200 increase 10/300GL column. **(D)** Negative-staining EM of the gE-NPs and naked NPs.

The extracellular domain of VZV gE (aa 31-544) (47) was engineered with a signal peptide for secretion from mammalian cells, and a C-terminal Tag from the isopeptide linkage system or the I53-50A component to enable antigen display on the corresponding NP carriers (**Figure 1A**). The gE-Tag and gE-I53-50A constructs were codon optimised and expressed in a stable Chinese hamster ovary (CHO) cell line as a secreted protein. After screening single-cell clones for the highest productivity, the selected monoclonal cell was cultivated in shake flasks using a 13-day fed-batch process. From the clarified supernatant, high-purity fusion proteins were produced using a combination of orthogonal chromatographic methods on AKTA systems (Cytiva) and buffer exchanged into the final formulation buffer (20 mM Tris-HCl, 150 mM NaCl, pH7.4) using a tangential flow filtration (TFF) system (Cobetter) equipped with Pellicon 2 Biomax 10 kDa MWCO membrane (Merck-Millipore). The Catcher from the isopeptide linkage system was genetically fused to a flexible linker followed by the subunit of the selected NP carriers, including a computationally designed NP, named NPM in this study, based on the 2-dehydro-3-deoxy-phosphogluconate (KDPG) aldolase as previously described (30), Ferritin NP (28), and AP205 VLP (48). This Catcher enables the formation of an isopeptide bond with the Tag (44). The final conjugated particles are named gEM, gE-Ferritin and gE-AP205 respectively. For gE-I53−50A, it self-assembles with the I53-50B component into a NP and is named gE-I53-50. These Catcher-NPs (NPM, Ferritin) or Catcher-VLP (AP205) were codon optimised and expressed in *Escherichia coli* BL21 (DE3). NPM was bulked up in an XDR-50 MO single-use fermenter (Cytiva), while other NPs were cultivated in 1 L shake flasks (NEST). All NPs were harvested in Sorvall Lynx 6000 centrifuges (Thermo Fisher Scientific). Intracellular proteins were first released using an AH-PILOT high-pressure homogeniser (ATS) and then clarified by a series of centrifugations. Cather-NPM was produced under the good manufacturing practice (GMP) compatible purification process as described before (30). Catcher- Ferritin and I53-50B were produced using prepacked HisTrap excel column (Cytiva) and Superdex 200 Increase 10/300 GL column (Cytiva) (buffer exchange into 20 mM Tris-HCl, 150mM NaCl, 25% sucrose w/v, pH 7.4 for catcher-Ferritin; and 50 mM Tris-HCl, 300mM NaCl, 0.75% CHAPS w/v, pH 7.4 for I53-50B) on an AKTA systems (Cytiva). Catcher-AP205 was purified using Ni-NTA resin (Qiagen), and buffer exchanged into 50 mM glycine, 25 mM sodium citrate, 0.1% (v/v) Tween 20, pH 8.0 through overnight dialysis at 4 °C. For conjugation, the catcher-NPM/Ferritin and gE-Tag were mixed at a 1:6 molar ratio and incubated for 24 h at 4°C, I53-50B and gE-I53-50A were mixed at a 3:1 mass ratio and incubated for 2 h at room temperature. Uncoupled gE-Tag and excessive I50-53B were removed from the conjugated NP by size-exclusion chromatography (SEC) using a Superdex 200 Increase 10/300 GL column (Cytiva) pre-equilibrated with 20 mM Tris-HCl, 25% sucrose w/v, pH 7.4 on the AKTA system (Cytiva). For gE-AP205, the catcher-AP205 and gE-Tag were mixed at a 1:2 molar ratio and incubated for 24 h at 22°C, then buffer exchanged into 50 mM glycine, 25 mM sodium citrate, 0.1% (v/v) Tween 20, pH 6.2 through overnight dialysis at room temperature. After separation, the conjugated NPs were analysed on sodium dodecyl sulfate polyacrylamide gel electrophoresis (SDS-PAGE) to determine the coupling efficiency by densitometry as previously described (30). The endotoxin levels of all NPs were all below 150 EU/mg.

### 2.2 Production of MF59-biosimilar adjuvant

MF59-biosimilar adjuvant was produced using a GMP-compatible method. Similar to the MF59 manufacturing, the process involves dispersing sorbitan trioleate in the squalene phase and polysorbate 80 in the aqueous phase, before high-speed mixing using the SMART LAB homogenizer (FLUKO) to form a coarse emulsion. The coarse emulsion then passed through a high-pressure ATS-AH pilot homogenizer (ATS engineering limited) to produce a fine emulsion. The emulsion is filtered through a sterilized filter, yielding MF59-bio at droplet size around 145-165nm.

### 2.3 SDS-PAGE

For SDS-PAGE, NuPAGE 4-12% Bis-Tris Midi Gel (Thermo Fisher Scientific) was used for all the analyses in this study. Before loading, protein samples were mixed with NuPAGE LDS Sample Buffer (Thermo Fisher Scientific) containing 5 mM DTT and denatured at 95°C for 5 minutes (min). Electrophoresis was performed at 150 V for 1 h in an Xcell4 SureLock Midi-Cell (Thermo Fisher Scientific) filled with 1× NuPAGE MES SDS Running Buffer (Thermo Fisher Scientific). Gels were then stained with InstaBlue Protein Stain Solution (APExBIO) and imaged using the Amersham ImageQuant 800 system (Cytiva).

### 2.4 BCA assay

Bicinchoninic acid (BCA) assay was performed using Pierce BCA Protein Assay Kit (Thermo Fisher Scientific) for total protein quantification according to the manufacturer’s instructions. Briefly, a series of bovine albumin standards were diluted to 0.025-1.5 mg/mL using the same diluent as the sample. 25 µL of each standard and sample were pipetted into a Nunc Microwell 96F plate (Thermo Fisher Scientific) in triplicate and incubated with working reagent at 37°C for 30 min. Absorbance at 562 nm was measured using an Infinite 200 PRO plate reader (Tecan) and plotted to construct a standard curve for sample evaluation.

### 2.5 Dynamic light scattering

Dynamic light scattering (DLS) was performed using Malvern Zetasizer Lab (Malvern) equipped with a 633 nm He-Ne laser. Size measurements were operated at an angle of 90° and data were collected and analysed using ZS XPLORER (Malvern). Approximately 1 mL of the sample (at 0.25 mg/mL) was measured in a DTS0012 disposable polystyrene cuvette (Malvern) at a controlled temperature of 25°C. Viscosity and refractive index at 25°C have been adjusted accordingly. Testing of each sample was repeated 3 times.

### 2.6 Negative staining electron microscopy

3-4 µL of gE-NP samples at a concentration of 100 ng/μL was adsorbed onto a glow-discharged carbon-coated copper grid for 1 min. The grid was then washed with Milli-Q water and blotted to dry. Negative staining was performed with 0.75% uranyl formate for 1 min. Electron micrographs of gE-NP were recorded using an FEI Tecnai 12 transmission electron microscope (Thermo Fisher Scientific) equipped with an Orius SC200 CCD camera operating at 120 kV.

### 2.7 Immunization

For naïve mice immunization, six to eight weeks female BALB/c mice purchased from Ruige biotech, housed in specific-pathogen-free (SPF) environments, were vaccinated, depending on the design of the experiment, with either 0.5µg or 5µg of vaccine (split over both legs) via the intramuscular (i.m.) route, using a prime-boost regime (prime on day 0 and boost on day 14). gE-NP immunogens were formulated with PBS, Alhydrogel (7.5μg per dose), or MF59-bio adjuvant (25 μL per dose) and mixed thoroughly before injection, 0.5µg or 5µg Shingrix (lyophilized gE antigen reconstituted in AS01B) was used as the control. The total injection volume for mice was 50μL. Blood samples were collected on days 14 and 28, and sera were obtained from whole blood by leaving samples overnight at 4°C to clot, followed by 10 min centrifugation at 16,000 × g at RT. Sera were pipetted into fresh Eppendorf tubes and frozen until further analysis. Spleen was harvested on D28 for Elispot assay.

For VZV LAV primed mice experiment, BALB/c mice were first immunized subcutaneously with 1/10 human dose (50μL) of VZV LAV vaccine (Varilrix™, GSK, not less than 10^3.3^ plaque-forming units (PFU) of the varicella-zoster virus (Oka-strain) per human dose) on Day-35. On Day 0 and Day 28, intramuscular injections of either 0.5μg of gEM/MF59-bio or 0.5μg of Shingrix were administered to the vaccine groups. The LAV-only group, that received one dose of LAV followed by two doses of MF59-bio and the adjuvant-only group, that received three doses of MF59-bio were included as controls. The total injection volume for all groups was 50μL. Sera and Spleen were harvested on Day 56 for ELISA, ELISPOT and intracellular cytokine staining (ICS) assays.

For VZV LAV primed NHP study, three to four years old male and female rhesus macaques were housed in a SPF environment and were first immunized subcutaneously with full human dose (0.5 mL) of VZV LAV vaccine (Varilrix™, GSK, not less than 10^3.3^ PFU of the varicella-zoster virus (Oka-strain) per human dose) on Day 1 followed by intramuscular injection of either 50μg of Shingrix (0.5 mL, full human dose) or 50μg of gEM (0.5 mL, formulated with MF59-biosimilar) on Day 50 and Day 78. Serum and PBMC were collected at the indicated time points, as described in the Results, for immunogenicity analysis.

### 2.8 Ethical statement

BALB/c mouse experiments complied with relevant ethical regulations regarding animal research. Protocols of mouse experiments for the immunization studies were approved by the Guangzhou Forevergen Biosciences Animal Experimentation Ethics Committee. Rhesus macaques were first housed in Medleader Bio-Pharm for immunization, in strict accordance with its Institutional Animal Care and Use Committee (IACUC) with the permit number: IACUC-2021-003.

### 2.9 IgG endpoint ELISA

Anti-VZV gE total IgG endpoint titer of serum collected from immunized animals was determined by indirect ELISA assay. 96-well Nunc MaxiSorp plates (Thermo Fisher Scientific) were coated with VZV gE at 100 ng/50 μL/well overnight at 4°C. After washing two times with 0.05% Tween 20 in PBS (PBST), the plates were blocked with Blocker Casein in PBS (Thermo Fisher Scientific) at 250 μL/well for 1 hour (h) at room temperature (RT) before washing two times again with PBST. Serially diluted sera were applied to each well for 1 h at RT, the plate was then washed four times with PBST followed by incubation with 1:5000 dilution of goat anti-mouse IgG conjugated with HRP (Abcam) or goat anti-monkey IgG conjugated with HRP (Abcam) for 1 h at RT. After a further six times wash with PBST, the plates were developed using Tetramethylbenzidine (TMB) (Thermo Fisher Scientific) for 10 min at RT. Subsequently, 1 N HCl was added to stop the reaction, the absorbance was recorded, and the value was read as 450 nm – 620 nm. The endpoint titer is defined as the X-axis intercept of the dilution curve at an absorbance value (+ three standard deviations or 0.15, whichever was higher) greater than the optical density (OD) for a naïve serum.

### 2.10 Fluorescent antibody to membrane antigen assay

The FAMA assay was performed as described previously (49). In brief, human fetal lung diploid fibroblasts (2BS) were infected with varicella zoster virus (attenuated Oka strain) and incubated for a time sufficient for VZV antigen expression on the cell surface, then cells were harvested by trypsin digestion until 50-75% of cells showed a cytopathic effect. The infected cells were resuspended in PBS and the cell density was adjusted to 1.5×10^5^ cells/mL, a volume of 20 μL per well added into 12-well slides (Matsunami). The cells were fixed with a precooled acetone solution. NHP serum was gradient diluted and added to the slide for a 30 min incubation at RT followed by washing 3 times (5 min each time) with PBS and finally air dry. FITC labelled goat anti-monkey IgG secondary antibody (Abcam) was added and incubated for 30 mins at 37°C. The slide was then washed 3 times with PBS, and examined by fluorescence microscopy (Olympus). The FAMA titer was measured as the highest serum dilution producing the characteristic ring-like cell surface fluorescence under microscopic examination.

### 2.11 ELISPOT assay

The assay was performed using either mouse or monkey IFN-γ and IL-2 ELISPOT kit (MabTech). In brief, the pretreated plates were washed with Dulbecco’s PBS (DPBS) and blocked with complete media for 30 min at RT. VZV gE peptides (aa 31-544, 20 aa in length with 10aa overlapping, synthesized by GenScript) were prepared and plated at 0.1-0.25µg per peptide per well. A total of 500,000 mouse splenocytes or 300,000 rhesus macaques PBMCs were added to each well and incubated at 37°C, 5% CO_2_ for 20 h. The next day, plates were washed with PBS, biotinylated mAb specific for IFN-γ and IL-2 (MabTech) were added in separate wells and incubated at RT for 2 h followed by wash and incubation with Streptavidin-ALP (MabTech) for an additional 1 h. A final wash step was followed by the addition of development solution BCIP/NBT-plus for 10 min. Plates were washed with water to stop development and dried before reading by ELISPOT reader (AID).

### 2.12 Intracellular cytokine staining and flow cytometry

For mice ICS study, single splenic cells (1 × 10^6^ cells) were isolated from immunized female BALB/c mice and restimulated *in vitro* over 6 h using a pool of 20-mer VZV-gE peptides (final concentration 1µg/mL) and CD28/CD49d Co-Stimulatory Antibodies (BD Biosciences). Cells were then incubated for 4 h with protein transport inhibitors (Brefeldin A; BD Biosciences) followed by washing with PBS and staining with the Fixable Viability Stain 780 (1:1000 final dilution; BD Biosciences) and Mouse Fc block (BD Biosciences) for 15 min. Cells were then washed with stain buffer and stained for 15 min with a mix of PE-Cyanine7 CD3 Monoclonal Antibody (1:50 final dilution; eBioscience), PE Rat anti-mouse CD4 (1:50 final dilution; BD Biosciences) and PerCP-Cy™5.5 Rat anti-mouse CD8 (1:50 final dilution; BD Biosciences) in a total volume of 50 µL. Cells were then fixed and permeabilized with Fixation/Permeabilization Solution Kit (BD Biosciences), washed twice with 1×Perm/Wash solution and stained with APC Rat anti-mouse IFN-γ (1:50 final dilution; BD Biosciences) as well as FITC Rat anti-mouse IL-2 (1:50 final dilution; BD Biosciences). Finally, cells were washed with 1×Perm/Wash solution, resuspended and analysed using a DxFLEX (BECKMAN COULTER). Live cells were gated (FSC/SSC) and acquisition was performed on ∼30,000 events. Data were analysed using CytExpert software (version 2.0.0.283). Data are represented as background subtracted from the mean responses of gE-specific CD4+ and CD8+ T cells, expressed as percentages of the total frequencies of CD4+ T cells expressing IFN-γ and IL-2.

For NHP, gE-specific CD4+ and CD8+ T cells expressing IFN-γ, IL-2 and/or TNF-α were detected using ICS and flow cytometry, as previously described (50). Single PBMC cells (1 × 10^6^ cells) were isolated from immunized rhesus macaques and restimulated *in vitro* for 2 h using a pool of 20-mer VZV-gE peptides (2.5 µg/mL) followed by further incubation for 14 h with protein transport inhibitors (Brefeldin A Solution; Biolegend). Cells were then washed with FCS (PBS containing 1% fetal calf serum) and stained with the Zombie NIR™ Fixable Viability Kit (BioLegend). Cells were further stained for 30 min with a mix of PerCP-conjugated CD3, BV421-conjugated anti-CD4 and FITC-conjugated anti-CD8 (1:100 final dilution; BD Biosciences) in a total volume of 50 µL. Cells were then fixed and permeabilized with Fixation/Permeabilization Solution Kit (BD Biosciences) and further stained with PE-Cy™7-conjugated anti-monkey IFN-γ, PE-conjugated anti-monkey IL-2 and BV650-conjugated anti-monkey TNF-α (1:100 final dilution; BD Biosciences). Cells were then washed twice with 1×Perm Wash solution, resuspended in FCS, and then analysed using a Cytoflex (BECKMAN COULTER). Live cells were gated (FSC/SSC) and acquisition was performed on ∼20,000 events. Data were analysed using FlowJo software (version 10.4.2). Data are represented as background subtracted from the mean responses of gE-specific CD4+ and CD8+ T cells, expressed as percentages of the total frequencies of CD4+ or CD8+T cells expressing IFN-γ, IL-2 and/or TNF-α.

### 2.13 Quantification and statistical analysis

Statistical significance was assigned when p-values were < 0.05 using Prism Version 9.0 software (GraphPad). Depending on the number of groups involved, statistical analysis was performed using either a Mann-Whitney test (to compare two groups of data) or the Kruskal Wallis test followed by Dunn’s multiple comparison post-test (to compare three or more groups of data). The statistical tests were performed in Prism 10 (GraphPad Software) and p-values less than 0.05 were considered significant. (* 0.01<p<0.05, ** 0.001<p<0.01, *** 0.0001<p<0.001, and **** p<0.0001).

## 3 Results

### 3.1 Development and characterization of the gE-conjugated nanoparticles

In the present study, we focused on the extracellular domain of VZV gE to design gE-displaying NP vaccines. gEM, gE-Ferritin and gE-AP205 were generated based on a split protein Tag/Catcher technology as previously described (30). gE-I53−50 was created using a self-assembling two-component nanoparticle system (45). All gE-NPs have been successfully developed and their characteristics are summarised in **Figure 1B**. gE and Tag fusion protein connected by a rigid linker (EAAAK)_3_ had a higher yield, and higher immunogenicity when conjugated to NPM NP carrier than no linker or flexible linker (GSSSS)_3_ design (**Supplementary Figure 1**), suggesting the arrangement and spacing of gE on the NP could influence the immunogenicity. Thus, the rigid linker (EAAAK)_3_ was selected in designing all NP constructs in our study. The purified gE fusion protein, naked NPs and conjugated gE-NPs were uniform and pure, demonstrated by a clear single band in reduced SDS-PAGE and a single major peak in the size exclusion chromatography (SEC) (**Figure 1C**; **Supplementary Figure 2A**). It is noted that not all the NP subunits can be conjugated to the gE-Tag using the Catcher/Tag system, likely due to steric hindrance on the particle’s surface. The overall conjugation efficiency for the gEM, gE-Ferritin and gE-AP205 was more than 70% by densitometry analysis. The assembly efficiency of gE-I53-50 is 100%, as the formation of the I53-50 nanoparticle requires the complete assembly of I53-50A and I53-50B together. We observed the structural characteristics of the gE-NP vaccine candidates using negative staining electron microscopy (EM). As shown in **Figure 1D**, all gE-NPs were visible on the surface of the monodispersed particles. The particle characteristics of naked NPs and gE-conjugated NPs were further measured by dynamic light scattering (DLS), whereby both presented a uniform distribution of particle sizes. The DLS results also indicated that the hydrodynamic diameter of the gE-conjugated NPs increased due to antigen conjugation or display (**Supplementary Figure 2B**).

### 3.2 Immunogenicity of gE-NPs in naïve mice

**Figure 2:**
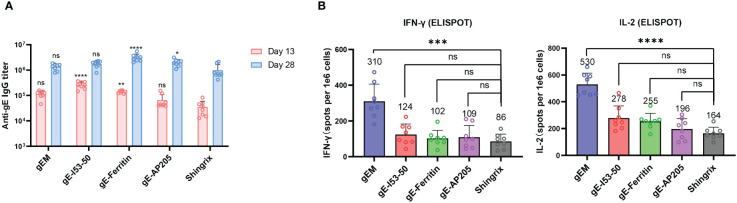
Immunogenicity of different gE-NP constructs in BALB/c mice. BALB/c mice (n=8 per group) were immunized intramuscularly twice with 5μg of different vaccine designs on day 0 and day 14 respectively, gEM, gE-I53-50, gE-Ferritin and gE-AP205 were formulated with MF59-biosimilar. Serum was collected on day 13 and day 28, spleen was collected on day 28 and processed to obtain the splenocytes. **(A)** Total IgG titers against antigen gE were measured by endpoint ELISA for day 13 and day 28 samples, GMT+95%CI is shown. Statistical analysis was performed between different gE-VLPs and Shringix at both D13 and D28. **(B)** ELISPOT was performed to measure the expression of IFN-γ and IL-2 from isolated splenocytes after stimulation with gE peptides, mean+SD is shown, the exact mean value is also displayed on the figure. One-way ANOVA with Dunns’ multiple comparison test was performed; ns, not significant; *p<0.05, **p<0.01 ***p<0.001, ****p<0.0001.

We compared the immunogenicity of the different gE-NPs with Shingrix. Naïve BALB/c mice (n=8) were immunized twice with 5 µg gEM, gE-I53−50, gE-ferritin or gE-AP205 formulated in MF59-bio and a 1/10 dose of Shingrix (5μg gE in 50μL AS01B) as a control on Day 0 and Day 14. The Sera of the immunized mice were collected on day 13 and day 28, and the spleen was collected on day 28. VZV-gE specific binding antibody titers were measured by endpoint ELISA. There is a trend towards higher antibody responses induced by gE-NPs than Shingrix after the prime and the boost vaccinations, although some are not statistically significant. (**Figure 2A**). We found no significant difference in the IgG titers between the gEM and Shingrix group after primary or boost immunization, while gE-ferritin induced significantly higher IgG titers than Shingrix after both primary and boost immunization. gE-I53−50 induced higher gE-specific IgG response compared to Shingrix only after primary immunization, and gE-AP205 showed higher gE-specific IgG titers than Shingrix after boost immunization. A robust VZV-specific cell-mediated immunity is required to prevent herpes zoster (51), thus we used ELISPOT assay to evaluate the induction of gE-specific splenocytes secreting IFN-γ or IL-2 by all vaccine candidates two weeks after boost immunization. Interestingly, the groups receiving gE-I53−50, gE-ferritin or gE-AP205, which demonstrated significantly higher IgG response than Shingrix, induced only similar levels of cellular immunity. gEM on the other hand demonstrated superior cellular immune response than Shingrix (**Figure 2B**). These results suggest gEM/MF59-bio is a promising shingles vaccine candidate.

### 3.3 Further evaluation of the immunogenicity of gEM in mice

**Figure 3:**
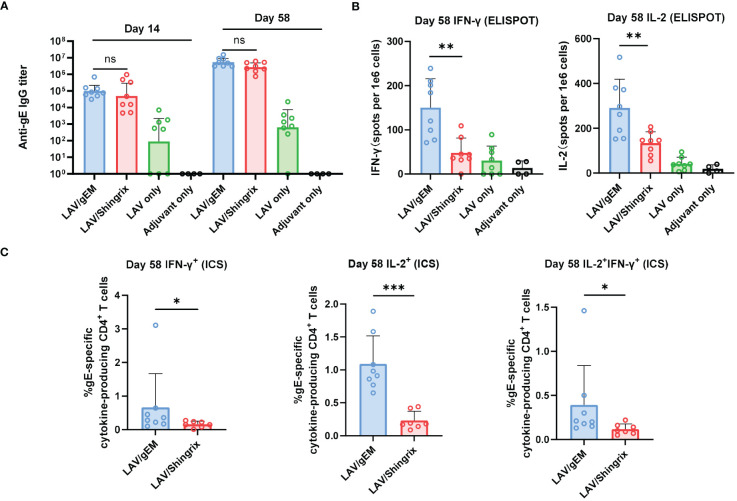
Immunogenicity of gEM after LAV vaccination in BALB/c mice. BALB/c mice (n=8 per group) were first immunized subcutaneously with live attenuated VZV vaccine on D-35 followed by intramuscular injection of 0.5μg of gEM (famulated with MF59-biosimilar), 0.5μg of Shingrix or adjuvant (LAV only group) on D0 and D28. Adjuvant only group that received three doses of MF59-biosimilar adjuvant was also included as a control. Serum was collected on day 14 and day 58. Spleen were harvest on day 58 and were processed to obtain splenocytes for ELISPOT. **(A)** Total IgG titers against antigen gE were measured by endpoint ELISA for each time-point, GMT+95%CI is shown. **(B)** ELISPOT was performed to measure the expression of IFN-γ and IL-2 from isolated splenocytes (Day 58) after stimulation with gE peptides, mean+SD is shown. **(C)** ICS was performed to measure the percentage of single or double cytokine secreting CD4+ splenocytes after gE peptides stimulation, mean+SD is shown. Mann-Whitney test was performed to compare gEM and Shingrix, ns = not significant, * p<0.05, ** p<0.01 *** p<0.001.

To further evaluate the immunogenicity and to find the best vaccination strategy for gEM, naïve BALB/c mice (n=6) were immunized twice intramuscularly with either 0.5μg or 5μg of gEM or Shingrix respectively, in which the gEM was formulated with PBS, Alhydrogel or MF59-bio. Both the gE-specific IgG titers and cellular immunity were measured. At both doses tested, the gEM/MF59-bio group induced the highest IgG response and cellular response compared to all other groups including Shingrix (**Supplementary Figure 3**), confirming the absolute importance of MF59-bio in gEM’s vaccine formulation and its subsequent immunogenicity. We next evaluated the immunogenicity of gEM under different vaccination regimes. Mice (n=6) were immunized twice intramuscularly with different doses of gEM (0.1μg, 1μg or 10μg) formulated with MF59-bio, and with different prime-boost intervals (14 days, 28 days or 56 days). The results showed the vaccination at intervals of 56 days induced the highest humoral and cellular immune response (**Supplementary Figure 4**). The majority of patients suffering from HZ are seropositive for varicella–zoster virus (52). Therefore, we need to evaluate the vaccine’s immunogenicity in VZV seropositive animals. Hence, BLAB/c mice (n=8) were first immunized subcutaneously with VZV LAV followed by two immunizations of 0.5 µg gEM/MF59-bio or 0.5μg Shingrix (0.5μg gE in 50μL AS01B) in a 28-day interval, five weeks after VZV LAV immunization. There was no significant difference in gE-specific IgG titers between the two vaccine groups after both primary and booster immunizations (**Figure 3A**). In contrast, gEM/MF59-bio induced a significantly higher cellular immune response than Shingrix, measured by IFN-γ and IL-2 ELISPOTs (**Figure 3B**). ICS was performed on processed splenocytes, the results were consistent with the ELISPOT data. gEM/MF59-bio induced significantly more gE-specific CD4+ single or double cytokine-secreting cells (**Figure 3C**; **Supplementary Figure 5**). However, we did not detect the gE-specific CD8+ single or double cytokine-secreting cells in all groups using ICS. These data further confirmed gEM/MF59- bio to be a better shingles vaccine at least in the mice experiments carried out in this study.

### 3.4 gEM induced stronger gE-specific CD4 T cell responses than Shingrix in NHP

**Figure 4:**
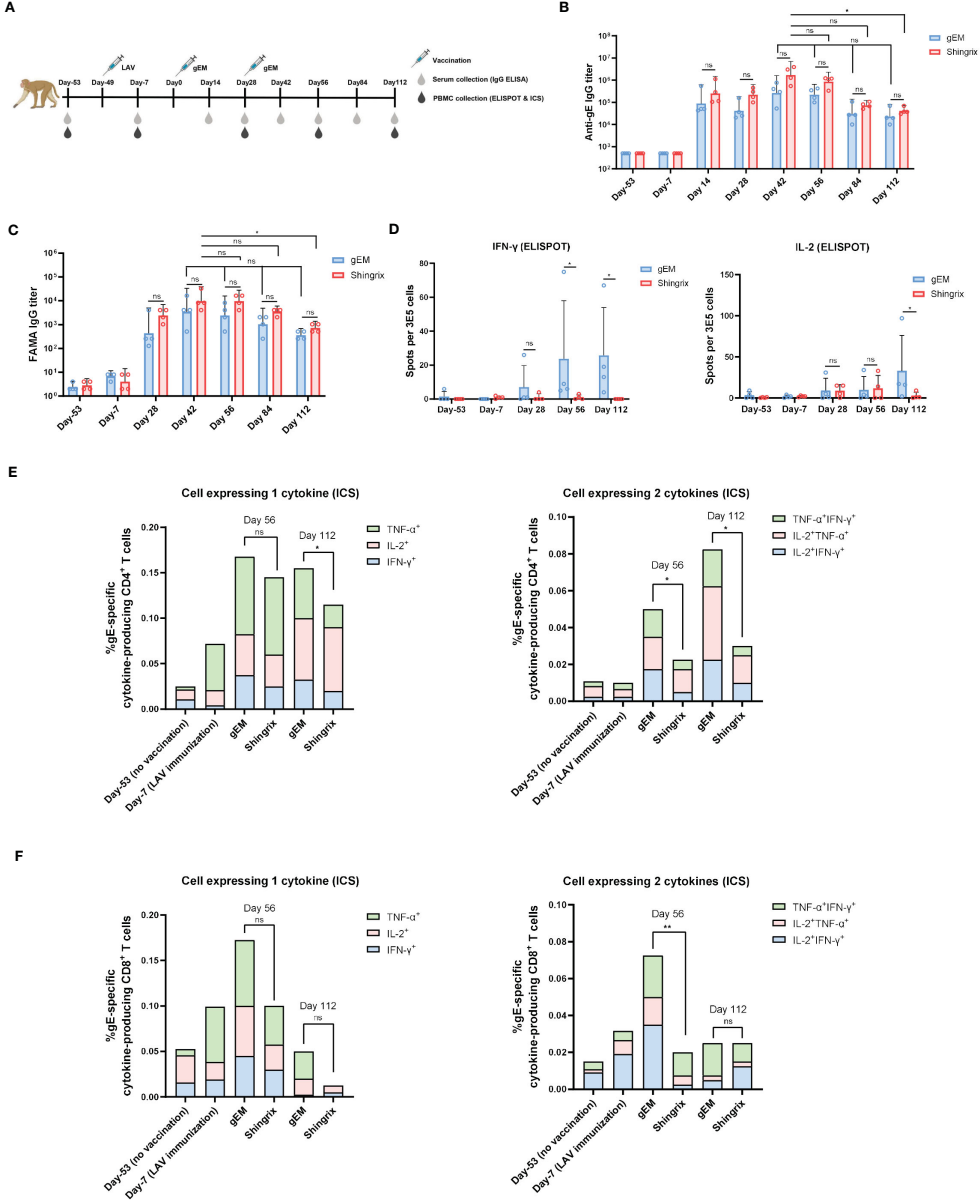
Immunogenicity of gEM in rhesus macaques. **(A)** Rhesus macaques (n=4 per group) were first immunized subcutaneously with live attenuated VZV vaccine on Day 1 (D1) followed by intramuscular injection of either 50μg of Shingrix or 50μg of gEM (famulated with MF59-biosimilar) on D50 and D78. Serum and PBMC were collected at the indicated time-points. **(B)** Total IgG titers against antigen gE were measured by endpoint ELISA for each time-point. **(C)** Functional antibody titers against natural viral proteins present on the surface of VZV-infected cells were measured by FAMA assay. **(D)** ELISPOT was performed to measure the expression of IFN-γ and IL-2 from isolated PBMCs after stimulation with gE peptides, mean+SD is shown. **(E, F)** ICS was performed to measure the percentage of single or double cytokine secreting CD4+ and CD8+ lymphocytes after gE peptides stimulation, mean value is shown. Mann-Whitney test was performed to compare gEM and Shingrix; ns, not significant; *p<0.05, **p<0.01.

To evaluate if the gEM vaccine is immunogenic in NHP, rhesus macaques (n=4) were first immunized subcutaneously with VZV LAV followed by two intramuscular injections of either 50μg of Shingrix or 50μg of gEM/MF59-bio in a 28-days interval, 50 days after VZV LAV prime (day 0). Humoral and cellular immune responses were assessed from sera and PBMCs were collected at the indicated time points (**Figure 4A**). The humoral response is defined by the gE-specific IgG titers, which are measured using gE endpoint ELISA. gE-specific IgGs were not detected after VZV LAV prime but were increased after each protein in adjuvant (PIA) boost, and both groups peaked after the second PIA boost with a similar trend. There was no significant difference in gE-specific IgG titers between the two vaccine groups. Although Shingrix showed a higher trend in maximal titers, it dropped sharply to similar levels as gEM on day 112 (**Figure 4B**). FAMA assay was also performed to measure the levels of antibodies that recognize natural viral proteins present on the surface of VZV-infected cells, which are reported as functional antibodies that correlate with protection from disease (53). The trend of the functional antibody response matched the gE-specific IgG titer measured by ELISA (**Figure 4C**). Consistent with mice studies, in NHP, gEM/MF59-bio also induced significantly higher levels of gE-specific IFN-γ secreting lymphocytes than Shingrix (from day 56), and higher gE-specific IL-2 response on day 112 (**Figure 4D**). To further evaluate the level of CD4+ T cell responses, which was demonstrated to be the key immunological marker for reduced HZ onset (11, 12), the percentage of single or double cytokine-secreting CD4+ lymphocytes was measured using intracellular cytokine staining (ICS) and flow cytometry. The gEM/MF59-bio induced a significantly higher percentage of gE-specific CD4+ T cells that secrete single or double cytokines, compared to those induced by Shingrix (**Figure 4E**; **Supplementary Figures 6A–C**). The percentage of double cytokine secreting CD8+ lymphocytes was also increased by gEM/MF59-bio vaccination on day 56 (**Figure 4F**; **Supplementary Figures 6D, E**). Collectively, these data demonstrated gEM/MF59-bio can induce superior CMI response in NHP as was in the mice model. It was confirmed in NHP, gEM/MF59-bio is a suitable shingles vaccine candidate.

## 4 Discussion

NP technology represents a new vaccine platform that has already shown promising results in both preclinical and clinical studies (54). We developed four new VZV vaccine candidates based on either the catcher/tag isopeptide linkage system to display VZV glycoprotein E (gE) on the surface of selected NP carriers or using the I53-50 two-component NP system to display gE antigen. The gE and NP carriers were produced with high purity, whilst the biophysical and structural analysis confirmed their quality. We then focused on identifying the most immunogenic vaccine candidate in terms of the cellular immune response. One of our main goals in developing the next-generation shingles vaccine is to reduce the demand for a potent adjuvant. We have selected and produced MF59-biosimilar as the adjuvant to test in our study. MF59 is well known for its safety profile (55) with a minimum (<1%) of grade 3 AE recorded in trials in children (56), as well as its ability to induce good cellular immune response demonstrated by influenza vaccine clinical trials in older adults (57). The main components of MF59 are Squalene, Span 85 and Tween 80, which are suitable for large-scale production without concerns of supply limitation as of AS01B in Shingrix.

We first tested vaccine candidates in a naïve mouse model, all four NPs exhibited a trend towards higher antibody and cellular response compared to the market vaccine control (**Figure 2**). gE-Ferritin/MF59-bio induced the highest antibody response, while gEM/MF59-bio demonstrated a remarkably higher cellular response revealed by ELISPOT assay. This observation suggests that different NP carriers could induce different levels of humoral and cellular immune responses. This could be due to the differences in the diameter of NP carriers, and the number or structure arrangement of displayed antigens, all of which may result in differences in the APC uptake and subsequent MHC presentations leading to T-cell stimulation or differences in the levels of B cell activation (58). Indeed, when gE-Tag was designed with either a flexible linker or rigid linker at its C terminus, after conjugation with NPM, their immune response increased dramatically compared to the design without a linker (**Supplementary Figure 1**), this is direct evidence demonstrating that antigen arrangement and surface spacing are key to the NP vaccine’s immunogenicity. Consistently, differences in functional antibody response were observed when comparing NP vaccines displaying antigen by chemical conjugation versus by catcher/tag system (59), showing immunogenicity is directly influenced by antigen arrangement on the surface of NP. Similarly, another study also observed differences in the antibody response when conjugating Influenza HA trimers, via the catcher/tag isopeptide linkage system, to AP205 or Mi3 carrier (60). All above could explain the differences we observed in the antibody response among all NP candidates. For cellular response, an early study discovered that 40-50 nm polystyrene beads conjugated with OVA stimulated more DCs in draining lymph nodes, subsequently inducing higher levels of IFN-γ response and antibodies compared to other sizes (20 nm or 100 nm) (61). A more recent study confirmed that the nanoparticle size determined the ability of particles to induce antigen-specific T CD8+ and Th1 responses and claimed 50 to 60 nm nanoparticles as the optimal inducers of CMI (62). We anticipate this size-dependent immune induction could be one of the many factors contributing to the differences in cellular response. The size of gEM is measured around 53 nm by DLS, which is the closest to the claimed optimal particle size compared to 65 nm, 34 nm and 40 nm of other tested NP vaccine candidates. Another study found CD4+ T cell epitopes in the ferritin NP contributed to the immune response towards the displayed antigen via ferritin-specific T follicular helper cells (Tfh) (63), suggesting the importance of the T cell epitopes within the NP. In addition, other biochemical properties of NP such as surface charge (64) and hydrophobicity (65), can influence the interactions with immune cells, resulting in enhanced immune activation. The properties mentioned vary among NP platforms and could lead to further differences in the cellular response. Additional studies are needed to compare the structural and biochemical characteristics of these NP platforms to confirm the underlying mechanisms behind the potential differences in immunogenicity.

For a good shingles vaccine candidate, the ability to elicit efficient VZV-specific CMI is critical to prevent latent VZV reactivation and HZ onset (15). From our initial study, gEM seemed to be the best candidate because it induced the highest CMI response among all NP vaccine candidates, and the CMI induced by gEM was even higher than the market vaccine control. Thus, further analysis was conducted on its formulation with different adjuvants including the classical adjuvant, Alum. As expected, the unadjuvanted gEM was poorly immunogenic, when it was formulated with alum, only a slight increase in antibody response was observed (**Supplementary Figure 3A**), and the level of IFNγ or IL-2 secreting splenocytes remained unchanged (**Supplementary Figure S3B**). This is in line with the fact that alum is a weak adjuvant and is biased to the T helper 2 (Th2) response (66). On the other hand, MF59 is known to induce a broader range of cytokines and chemokines than alum, thereby inducing a balanced Th1/Th2 response (67). As a result, gEM/MF59-bio induced higher antibody and cellular responses than other groups including Shingrix in both low and high dosages tested (**Supplementary Figure 3**). It is evident from our second study that oil-in-water adjuvants such as MF59 are required to maximize the immunogenicity of gEM, leading to a higher level of CMI response than the market vaccine. We have therefore decided the final formulation of our shingles vaccine to be gEM/MF59-bio. As for the vaccination regime, we compared different doses at a prime-boost interval of 14 days, 28 days and 56 days in the naïve BALB/c mice model. An interval of 56 days induced the highest humoral and cellular immunity in all three doses tested (**Supplementary Figure 4**), this may be due to a higher memory response from a delayed boost (68).

The VZV LAV-primed mouse (50) and NHP (15) models have been previously reported for assessing shingles vaccine. We have repeated these studies to further assess our vaccine candidate gEM/MF59-bio. In VZV LAV primed mice, similar to the result observed in a naïve mouse model, gEM/MF59-bio induced significantly higher cellular response than Shingrix, the antibody response on the other hand remained at similar levels (**Figure 3**). In the VZV LAV primed rhesus macaque model, there was no significant difference in gE-specific IgG titers (measured by ELISA) or functional antibody titers (measured by FAMA assay) between the two groups, the Shingrix group exhibited a higher trend in maximal titers; however, it could not be sustained and dropped to a similar level as that of the gEM over time after the final immunization (**Figures 4B, C**). This is consistent with the fact that the NP vaccine can induce more robust and durable humoral immunity than soluble protein. After immunization, NPs are preferentially trafficked to lymph nodes (LN) for antigen presentation and immune system activation (69), the NP could stay relatively longer in the LN and continuously stimulate the humoral immune responses (70, 71). For cellular immune response analysis, ELISPOT was first performed and confirmed the vaccine could induce a higher cellular immune response than Shingrix in NHP (**Figure 4D**). The level of CD4 T cells with Th1 phenotype was reported to correlate with protective effects on HZ or associated PHN (11, 12). Although not as important as the CD4 T cells, some studies also suggested that VZV-specific CD8 T cell response may also play a role in preventing VZV viral reactivation (72). In our study, gEM/MF59-bio induced significantly higher VZV-specific CD4 T cell responses and a higher CD8 T cell response than Shingrix (**Figures 4E, F**). This is not surprising as a clinical trial on a plant-derived VLP displaying influenza HA antigen has already demonstrated the VLP vaccine could elicit a higher CD4+ T cell response as well as a comparable CD8+ T cell response than a commercial inactivated vaccine (73).

In summary, we have compared the immunogenicity of four different NP vaccine candidates with the commercial vaccine Shingrix. gEM elicited a durable humoral immune response and the highest CMI among all candidates in mice. Because of the NP platform, safe and more accessible adjuvants such as MF59-bio can be used while the vaccine still maintains stronger immunogenicity than the best commercial vaccine. After further confirmation in rhesus macaques, gEM formulated in MF59-bio is proven to be a suitable next-generation shingles vaccine candidate for further evaluation. It is also worth noting that gEM is currently the only reported shingles vaccine under development that utilises NP platform technology to display the VZV gE antigen.

## Data availability statement

The original contributions presented in the study are included in the article/**Supplementary Material**. Further inquiries can be directed to the corresponding author.

## Ethics statement

The animal study was approved by Guangzhou Forevergen Biosciences Animal Experimentation Ethics Committee. Rhesus macaques were first housed in Medleader Bio-Pharm for immunization, in strict accordance with its Institutional Animal Care and Use Committee (IACUC) with the permit number: IACUC-2021-003. The study was conducted in accordance with the local legislation and institutional requirements.

## Author contributions

YYL: Conceptualization, Data curation, Formal analysis, Investigation, Project administration, Supervision, Validation, Writing – original draft. ST: Data curation, Formal analysis, Investigation, Methodology, Software, Validation, Writing – original draft. YA: Data curation, Methodology, Writing – original draft. ZH: Data curation, Methodology, Writing – review & editing. CM: Data curation, Methodology, Writing – review & editing. MF: Data curation, Formal analysis, Methodology, Writing – review & editing. ZX: Data curation, Formal analysis, Methodology, Writing – review & editing. YL: Data curation, Formal analysis, Methodology, Software, Writing – review & editing. SL: Data curation, Formal analysis, Methodology, Software, Writing – review & editing. YJZ: Data curation, Formal analysis, Methodology, Writing – review & editing. YZ: Conceptualization, Data curation, Methodology, Project administration, Supervision, Validation, Writing – review & editing. JJ: Conceptualization, Data curation, Formal analysis, Funding acquisition, Methodology, Project administration, Supervision, Validation, Writing – review & editing.
