# Supplementary material for: A nanoparticle vaccine displaying varicella-zoster virus gE antigen induces a superior cellular immune response than a licensed vaccine in mice and non-human primates
Source: Front Immunol. 2024 Jul 16;15:1419634. doi: 10.3389/fimmu.2024.1419634 (PMC11286566; doi:10.3389/fimmu.2024.1419634)
Supplement: Supplementary file 1 [file Presentation_1.pptx]

## Slide 1
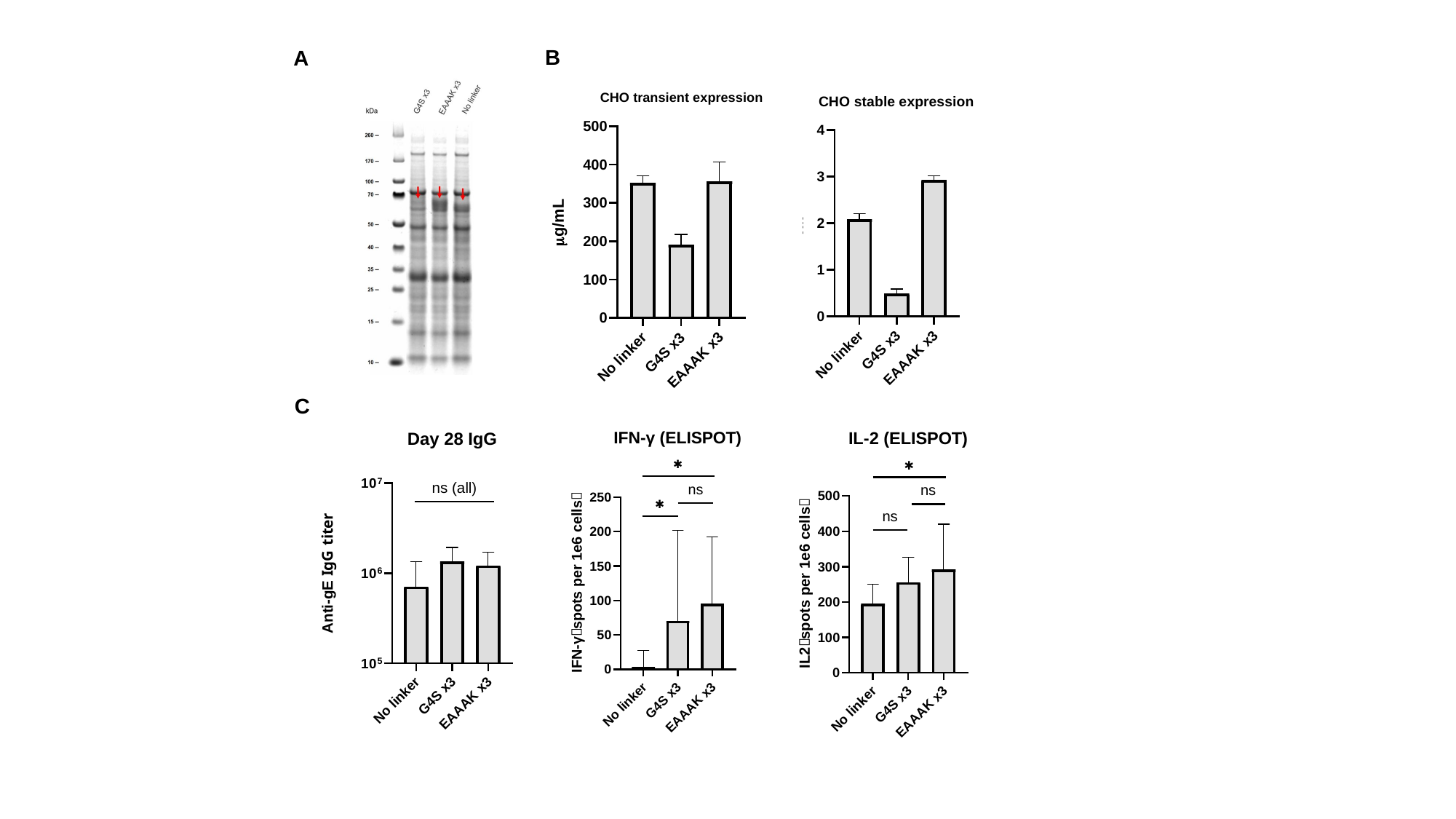

B
A
C

## Slide 2
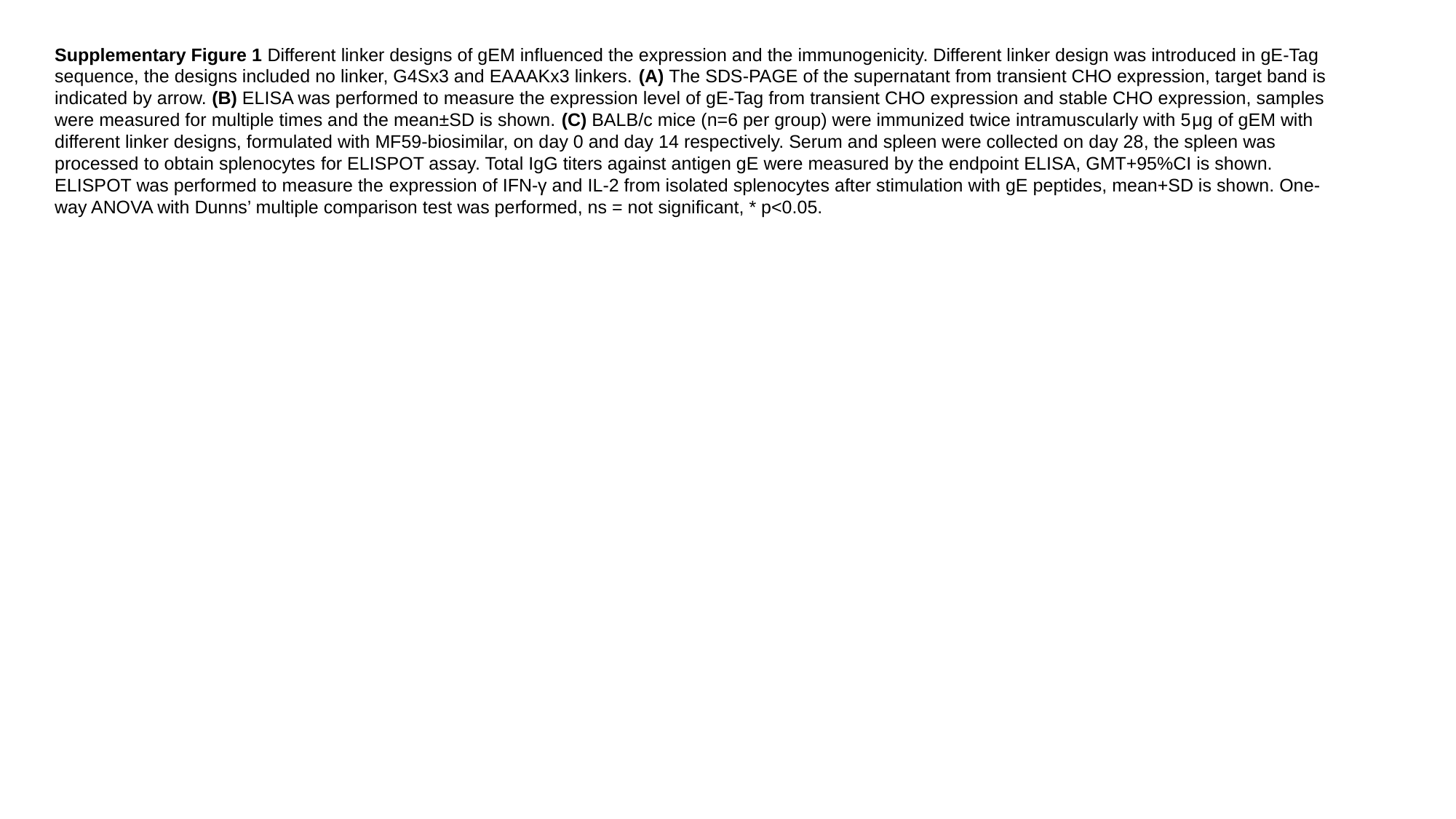

Supplementary Figure 1 Different linker designs of gEM influenced the expression and the immunogenicity. Different linker design was introduced in gE-Tag sequence, the designs included no linker, G4Sx3 and EAAAKx3 linkers. (A) The SDS-PAGE of the supernatant from transient CHO expression, target band is indicated by arrow. (B) ELISA was performed to measure the expression level of gE-Tag from transient CHO expression and stable CHO expression, samples were measured for multiple times and the mean±SD is shown. (C) BALB/c mice (n=6 per group) were immunized twice intramuscularly with 5μg of gEM with different linker designs, formulated with MF59-biosimilar, on day 0 and day 14 respectively. Serum and spleen were collected on day 28, the spleen was processed to obtain splenocytes for ELISPOT assay. Total IgG titers against antigen gE were measured by the endpoint ELISA, GMT+95%CI is shown. ELISPOT was performed to measure the expression of IFN-γ and IL-2 from isolated splenocytes after stimulation with gE peptides, mean+SD is shown. One-way ANOVA with Dunns’ multiple comparison test was performed, ns = not significant, * p<0.05.

## Slide 3
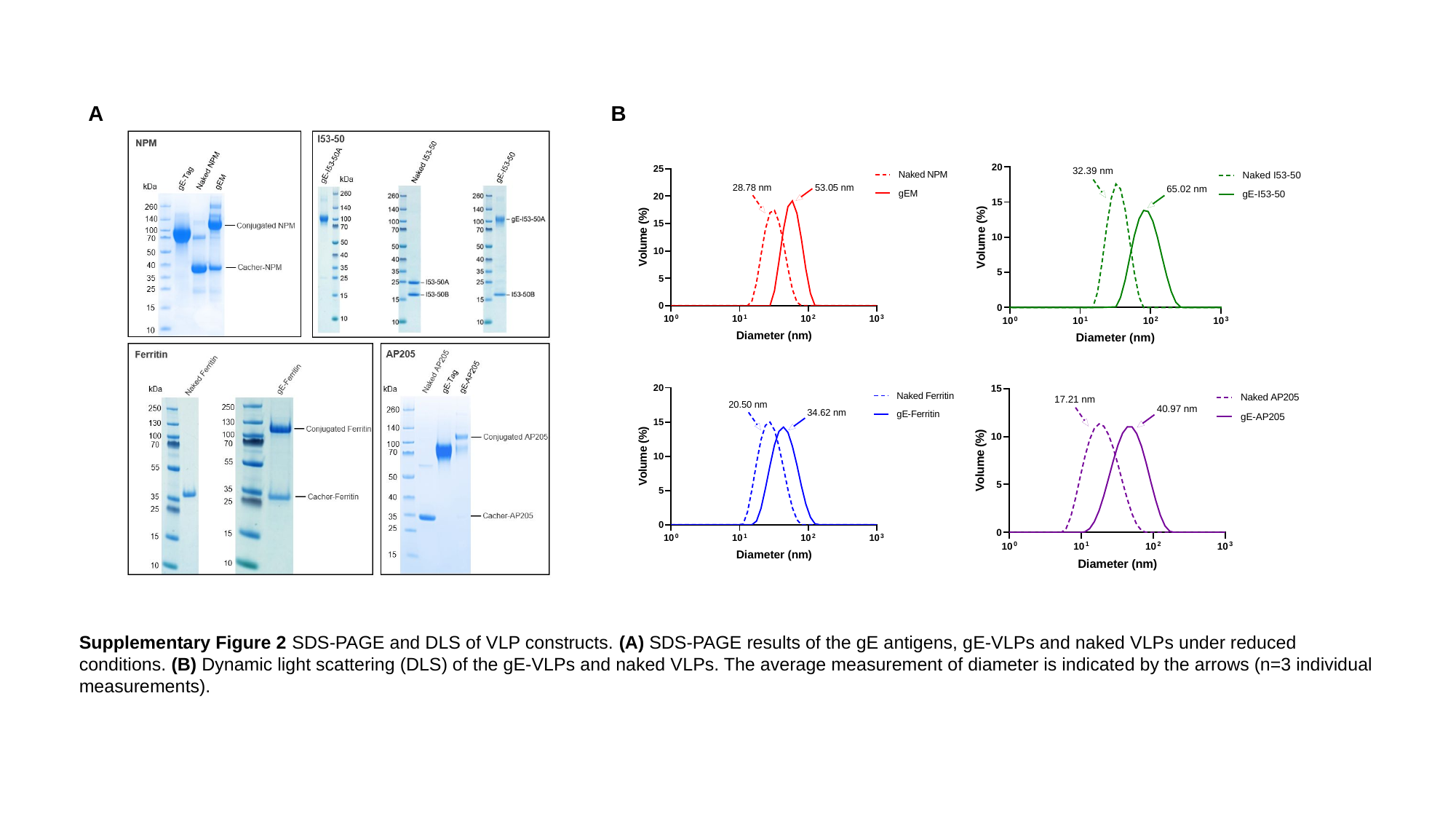

A
B
Supplementary Figure 2 SDS-PAGE and DLS of VLP constructs. (A) SDS-PAGE results of the gE antigens, gE-VLPs and naked VLPs under reduced conditions. (B) Dynamic light scattering (DLS) of the gE-VLPs and naked VLPs. The average measurement of diameter is indicated by the arrows (n=3 individual measurements).

## Slide 4
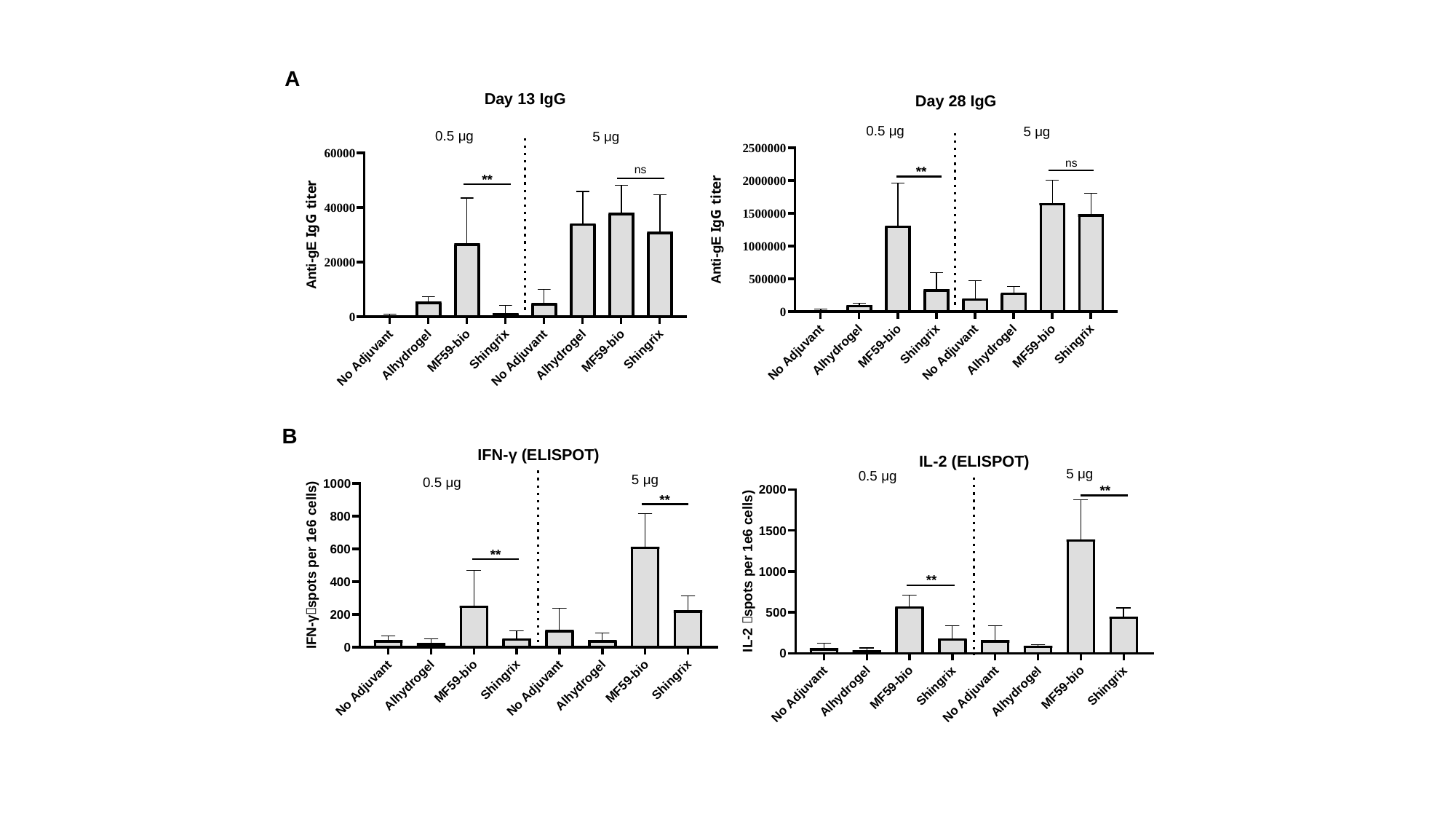

A
B

## Slide 5
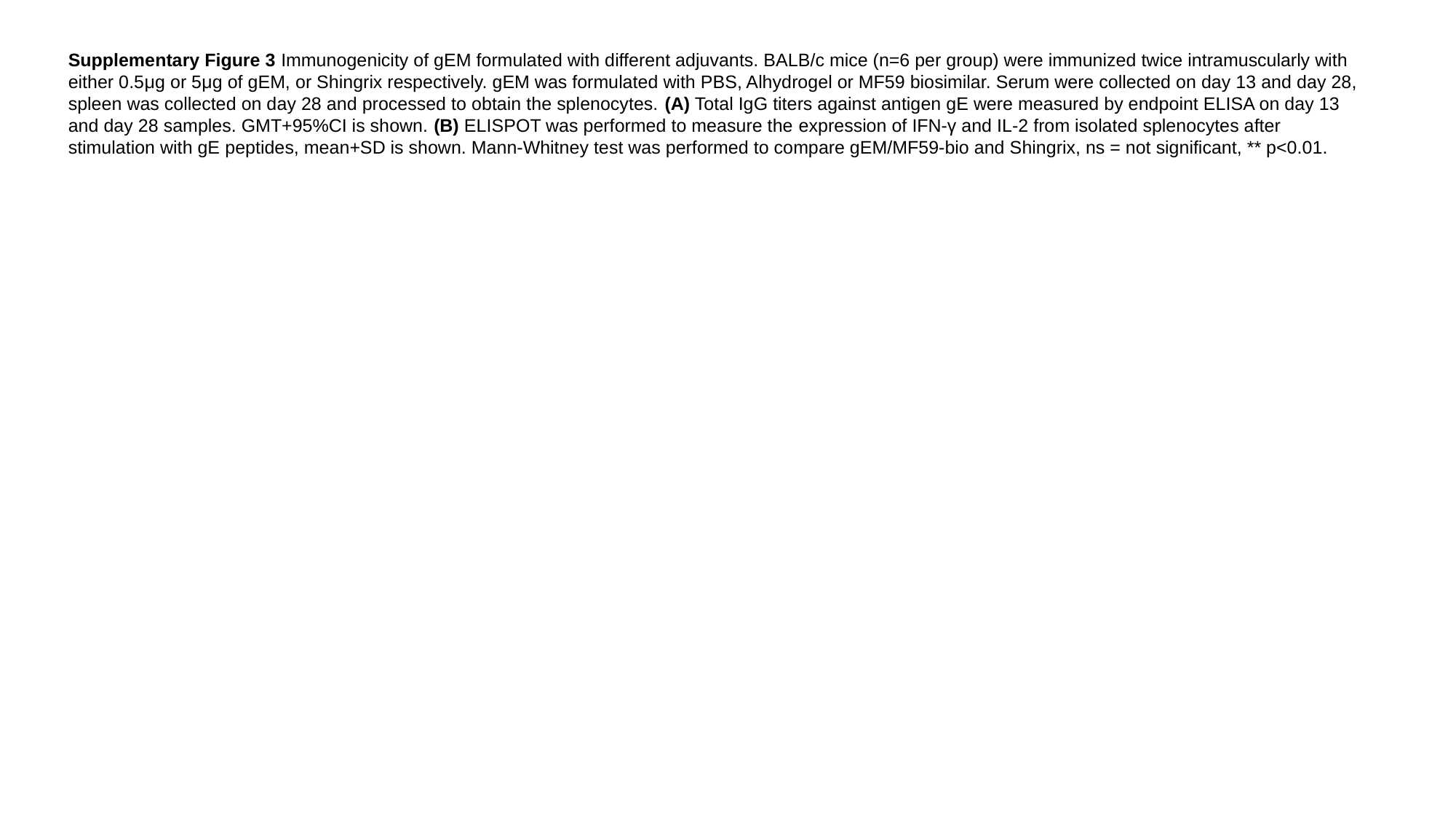

Supplementary Figure 3 Immunogenicity of gEM formulated with different adjuvants. BALB/c mice (n=6 per group) were immunized twice intramuscularly with either 0.5μg or 5μg of gEM, or Shingrix respectively. gEM was formulated with PBS, Alhydrogel or MF59 biosimilar. Serum were collected on day 13 and day 28, spleen was collected on day 28 and processed to obtain the splenocytes. (A) Total IgG titers against antigen gE were measured by endpoint ELISA on day 13 and day 28 samples. GMT+95%CI is shown. (B) ELISPOT was performed to measure the expression of IFN-γ and IL-2 from isolated splenocytes after stimulation with gE peptides, mean+SD is shown. Mann-Whitney test was performed to compare gEM/MF59-bio and Shingrix, ns = not significant, ** p<0.01.

## Slide 6
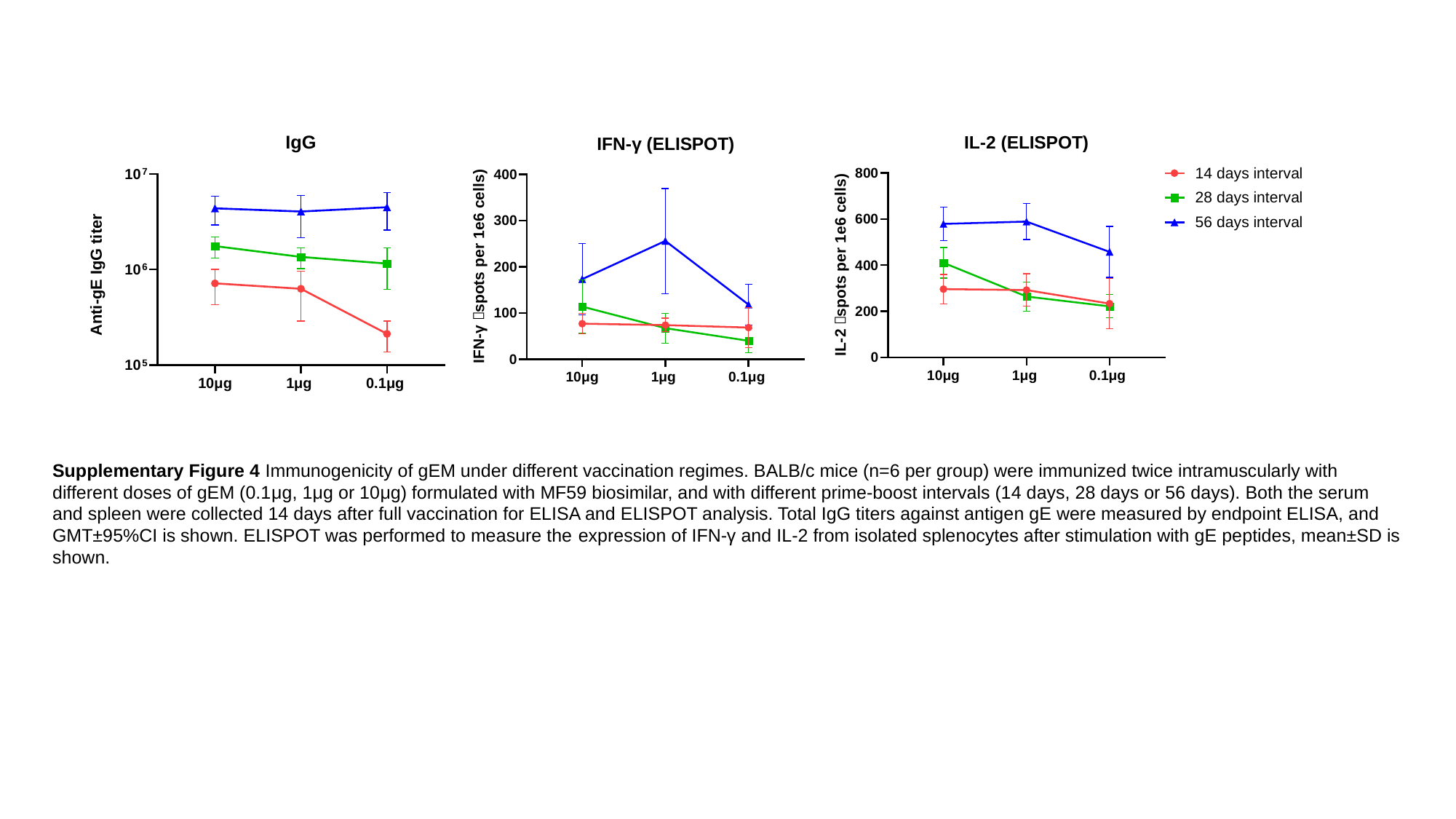

Supplementary Figure 4 Immunogenicity of gEM under different vaccination regimes. BALB/c mice (n=6 per group) were immunized twice intramuscularly with different doses of gEM (0.1μg, 1μg or 10μg) formulated with MF59 biosimilar, and with different prime-boost intervals (14 days, 28 days or 56 days). Both the serum and spleen were collected 14 days after full vaccination for ELISA and ELISPOT analysis. Total IgG titers against antigen gE were measured by endpoint ELISA, and GMT±95%CI is shown. ELISPOT was performed to measure the expression of IFN-γ and IL-2 from isolated splenocytes after stimulation with gE peptides, mean±SD is shown.

## Slide 7
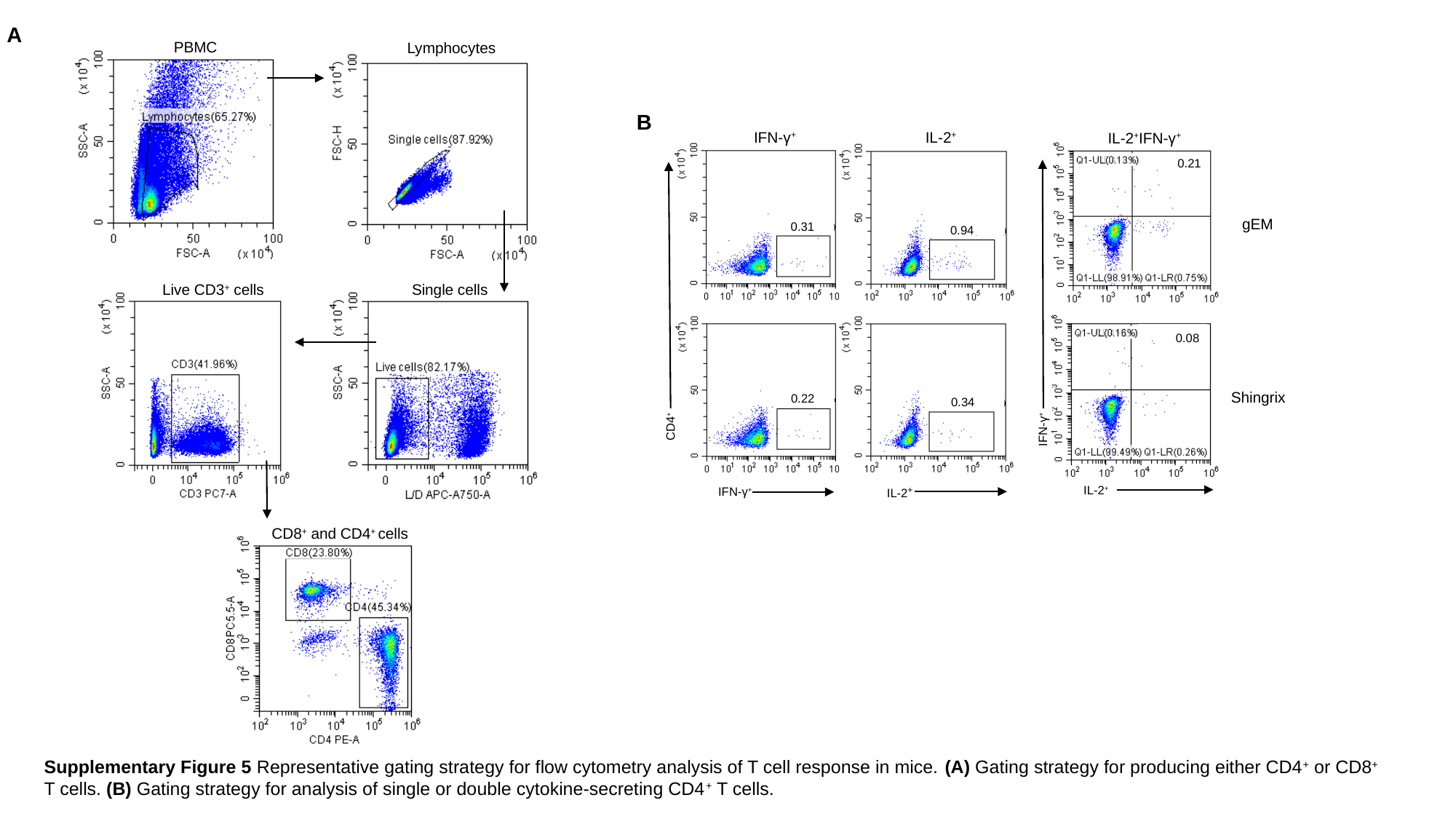

A
PBMC
Lymphocytes
B
IFN-γ+
IL-2+
IL-2+IFN-γ+
0.21
gEM
0.31
0.94
Live CD3+ cells
Single cells
0.08
CD4+
IFN-γ+
Shingrix
0.22
0.34
IL-2+
IL-2+
IFN-γ+
CD8+ and CD4+ cells
Supplementary Figure 5 Representative gating strategy for flow cytometry analysis of T cell response in mice. (A) Gating strategy for producing either CD4+ or CD8+ T cells. (B) Gating strategy for analysis of single or double cytokine-secreting CD4+ T cells.

## Slide 8
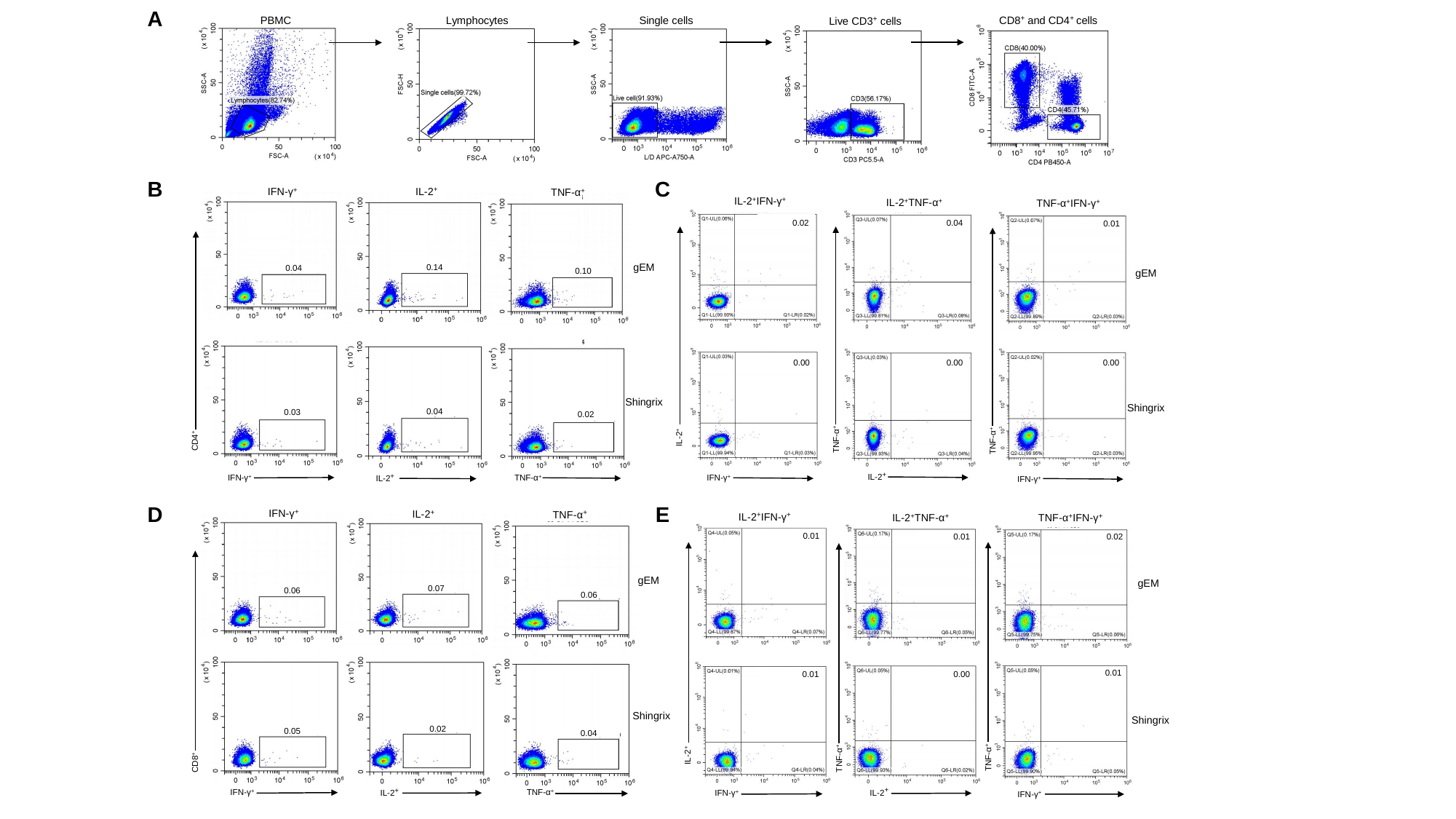

A
C
B
D
E

## Slide 9
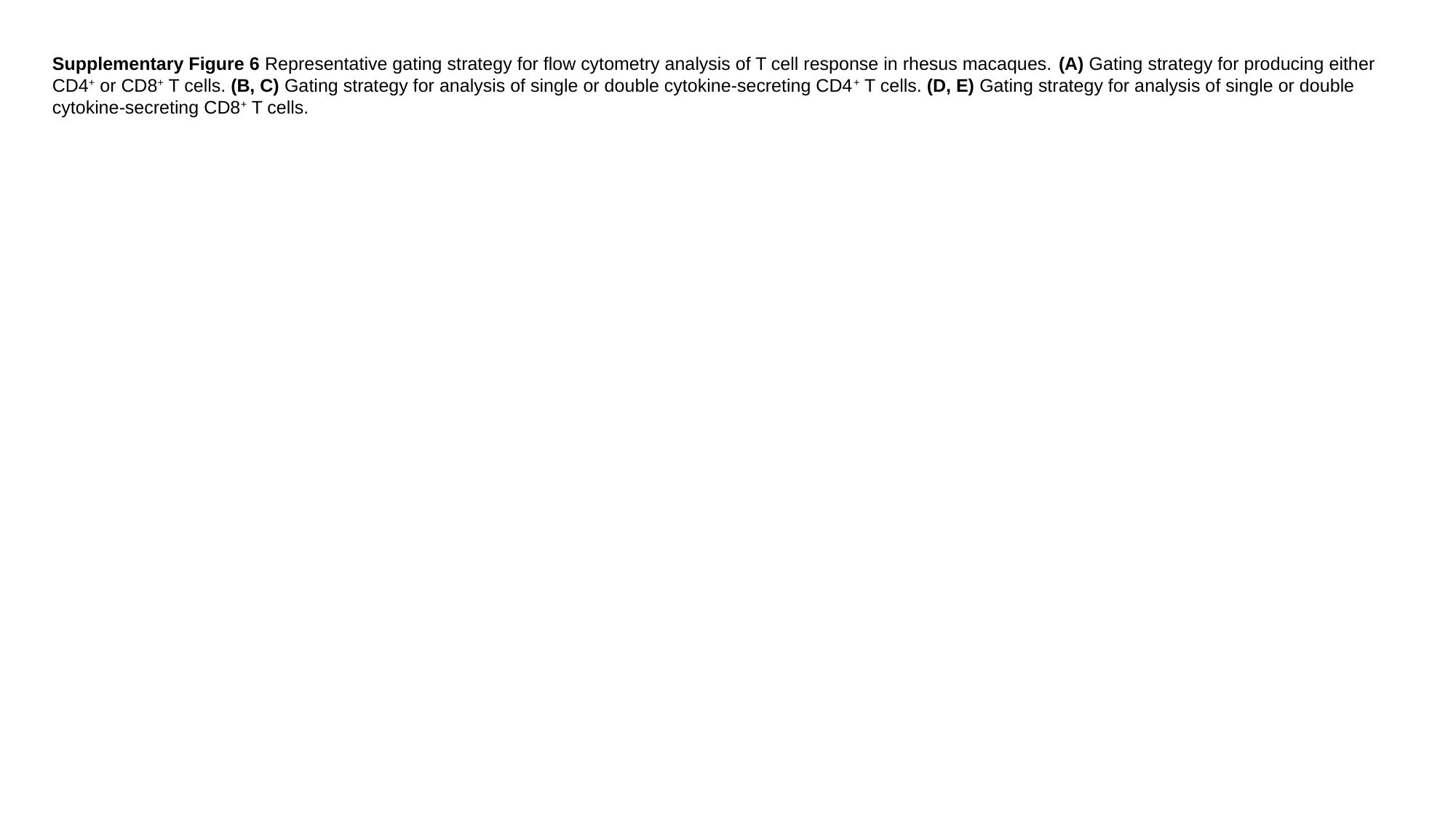

Supplementary Figure 6 Representative gating strategy for flow cytometry analysis of T cell response in rhesus macaques. (A) Gating strategy for producing either CD4+ or CD8+ T cells. (B, C) Gating strategy for analysis of single or double cytokine-secreting CD4+ T cells. (D, E) Gating strategy for analysis of single or double cytokine-secreting CD8+ T cells.
